# Case Report: Metastatic renal cell carcinoma to the thyroid— A rare encounter

**DOI:** 10.3389/fsurg.2022.1000425

**Published:** 2022-11-15

**Authors:** Megan Shepherd, Justin Lohmann, Laurentia Nodit, Tanaz Vaghaiwalla, Matthew Mancini

**Affiliations:** Departments of Surgery and Pathology, The University of Tennessee, Knoxville, TN, United States

**Keywords:** metastatic RCC, thyroid, cancer, endocrinology, oncology

## Abstract

**Background:**

Renal cell carcinoma (RCC) accounts for approximately 4% of new adult cancers. By fine needle aspiration, identification of metastatic RCC to thyroid is challenging; therefore, surgical resection is indicated for definitive characterization. Our report surveys metastatic RCC to thyroid in our hospital.

**Methods:**

Twenty years retrospective review of electronic records in our institution identified five patients with metastatic renal cell carcinoma to the thyroid. We analyzed patient charts and pathology reports to evaluate clinical parameters and therapy.

**Results:**

In all cases, the original RCC was of clear cell type. Pathologic tumor stage ranged from pT1a to pT3a, Fuhrman nuclear grade varied from 2 to 4 and angiolymphatic invasion was noted in one case. In three patients, RCC in the thyroid occurred as an oligometastasis with no evidence of disease in the nephrectomy bed or other parts of the body. In two patients, concomitant recurrent RCC and metastases to liver, lung, brain and chest wall were documented. The thyroid metastases were found approximately 10 years after completion of nephrectomy with a range of 0–21 years. Three thyroid fine needle aspirations correctly identified the disease, one was negative, and one was classified as atypical cells present, suspicious for RCC.

**Conclusion:**

The thyroid gland is an uncommon location for RCC metastasis and can appear across a wide range of initial stages and grades of the disease. Thyroid metastases occurred as late as 21 years from the initial tumor resection. Increased awareness and a high index of suspicion are needed to detect metastasis, as they can be found in atypical locations and mimic primary disease.

## Introduction

Renal cell carcinoma (RCC) is the dominant type of malignant neoplasm arising from the kidneys, responsible for more than 14,000 deaths per year (1, 2). It is often detected late due to lack of symptoms with 20% of patients already metastatic at the time of diagnosis (2). RCC more commonly affects males, with a peak incidence between the ages of 50–70 years. The thyroid is found to contain metastatic disease in 1.4%–3% of patients that undergo surgery for thyroid neoplasm (3) and in 1.25%–24% in autopsy series (4). Due to significant morphologic overlap, it can be extremely difficult to identify metastases by review of fine needle aspiration alone and some metastases can be misdiagnosed as a primary thyroid neoplasm. Therefore, surgical resection is indicated for definitive characterization. Our report reviews five cases of metastatic RCC to the thyroid in our hospital.

## Methods

This study was a retrospective review of the electronic health record over a 20-year period at a single institution. Five patients were found and noted to have metastatic renal cell carcinoma of the thyroid. We reviewed their records looking especially at pathology records, age of diagnosis of primary cancer, and time to metastatic disease. We also determined their treatment plan including the time of each surgery and what chemotherapy regimen they underwent.

## Results

Our study found five patients with renal cell carcinoma metastatic to the thyroid. There were three females and two males, and the time from primary tumor identification until the diagnosis of metastasis ranged from 0 to 21 years, as noted in Table 1. One of the patients in the study was found to have thyroid metastasis at the time of their renal cell carcinoma diagnosis. Their initial presentation stage ranged from pT1a to pT3a. One of the patients was noted to have lymphatic invasion. In three patients, RCC in the thyroid occurred as an oligometastasis with no evidence of disease in the nephrectomy bed or other parts of the body. In two patients, concomitant recurrent RCC and metastases to the liver, lung, brain, and chest wall were documented.

**Table 1:** Characteristics of the original RCC and the timing of metastasis to the thyroid.

Sex	Date of kidney resection	Stage of kidney cancer	Date of thyroid biopsy	Time from resection to thyroid metastasis (years)
F	1999	pT2a, 7.8 × 7.6 cm, mixed clear and granular cell, grade 4	2020	21
F	2005	pT1a, 2.9 cm, grade 2	2018	13
M	2009	pT2N0, 7.5 cm, grade 2-3,	2015	6
M	2010	pT3a, 10.5 × 8 × 6.5 cm, grade 3, tumor through capsule	2010	0
F	1999	Unknown	2000	1

Three thyroid fine needle aspirations (FNAs) correctly identified the disease, one was negative, and one was classified as atypical cells present, suspicious for RCC. Three patients underwent chemotherapy, all initially with sunitinib after nephrectomy. One of the three initially refused chemotherapy but was started on sunitinib after they were noted to have progression of his disease. The last patient had their primary tumor at an outside hospital and no records noted if they received any treatment at that time.

Our patient is an 81-year-old female was referred to our clinic by her endocrinologist after noticing that her right thyroid nodule had increased in size over the last 6 months. She has a medical history of renal cell carcinoma, hyperlipidemia, and type 2 diabetes. She had previously undergone an L nephrectomy in 1999 and a hysterectomy in 1990. She denied any previous use of tobacco, alcohol, or illicit drugs. She had a family history of stomach cancer in her mother.

The original pathological diagnosis was mixed clear/granular cell adenocarcinoma, which is now labeled clear cell renal cell carcinoma (CC-RCC). At the time of diagnosis, the tumor was confined to the kidney, without angiolymphatic invasion. The nuclei were given a Fuhrman grade 4 due to the bizarre and often multinucleated nuclei, with heavy chromatin clumps and prominent nucleoli. One lymph node was examined and found to be negative for malignancy. Because the tumor was 7.8 cm in greatest dimension and limited to the kidney, it was a pathologic stage pT2a. In November 2016, the patient underwent a fine needle aspiration of her right thyroid. The specimen was adequate with follicular epithelial cells and scattered colloid, consistent with a benign follicular nodule. There was no evidence of malignancy; however, sampling bias is always an issue.

With the recently reported increase in size, a repeat ultrasound was completed by her endocrinologist, and she was found to have a large right-sided nodule that was hypervascular measured 5.7 × 3.4 × 4.5 cm, and had increased in size from her previous ultrasound in 2018. She also noted occasional dysphagia. Her thyroid function testing reported normal levels of thyroid stimulating hormone and thyroxine. She denies any hoarseness or difficulty breathing. She was notified during the examination that she has a large nodule within the right thyroid that was irregular but without tenderness.

**Figure 1:**
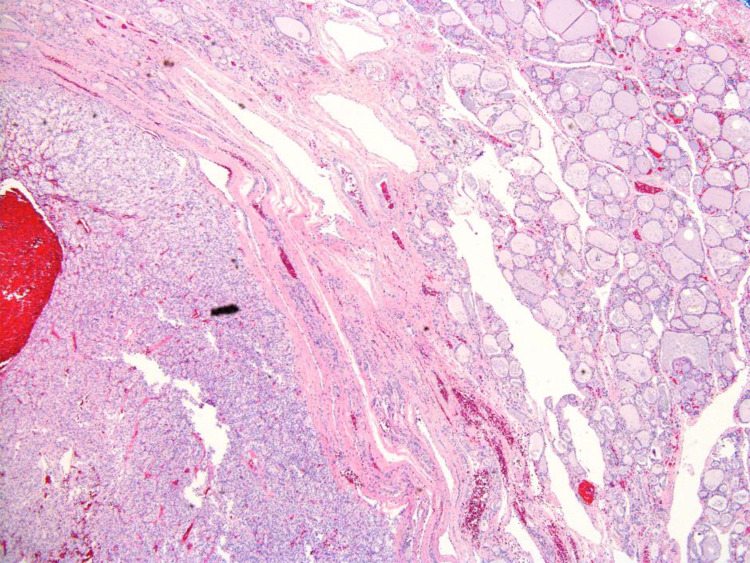
Renal cell carcinoma, clear cell type (lower left corner), benign thyroid parenchyma upper right corner, microscopic photograph, 200× magnification, hematoxylin and eosin stain.

**Figure 2:**
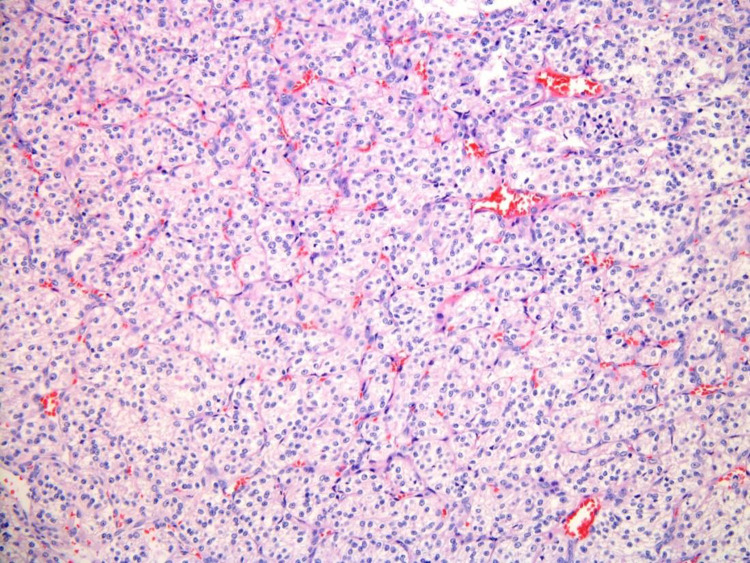
Renal cell carcinoma, clear cell type, microscopic photograph, 200× magnification, hematoxylin and eosin stain.

The patient underwent a right-lobe partial thyroidectomy in June 2020. The history of CC-RCC was noted in previous pathology reports. The gross examination of the resected specimen demonstrated a “well-circumscribed golden-yellow-tan mass” in which there were areas of hemorrhage present. Histologic sections showed a partially encapsulated, nodular proliferation composed of monotonous, intermediate-sized cells, embedded within benign thyroid parenchyma (Figure 1). At higher power examination, the mass is composed of clear cells with centrally located nuclei, prominent nuclei, and abundant vascular capillary network (“chicken wire”) characteristic of clear cell renal cell carcinoma (Figure 2). Immunohistochemical stains were performed to further evaluate the site of origin for the neoplastic cells. The renal markers PAX8 and RCC were positive, while the parathyroid and thyroid markers (PTH and TTF1) were negative. Pancytokeratin was focally reactive and the neuroendocrine marker synaptophysin was negative excluding an endocrine neoplasm. The morphologic and immunohistochemical features of this neoplasm, along with her known history, were most consistent with metastatic clear cell renal cell carcinoma.

The patient's PET/CT scan did not demonstrate any signs of residual or metastatic disease. Due to her advanced age and the 21 years between her initial disease and metastatic disease, our multidisciplinary tumor board elected to observe the patient with repeat PET in 4 months and not initiate chemotherapy at this time.

## Discussion

Renal cell carcinoma treatment is performed primarily by surgical resection with stages I–III successfully removed by partial or radical nephrectomy (2). For patients with metastatic disease, the therapeutic options are more limited as renal cell carcinoma is generally not sensitive to radiation or cytotoxic chemotherapy agents (2). The first line of treatment in metastatic clear cell RCC is now sunitinib malate, an oral multitargeted tyrosine kinase receptor inhibitor with antitumor and antiangiogenic activities, which block VEGFR, KIT, and PDGFR in both biochemical and cellular assays (2).

Three of the five of our patients underwent treatment with sunitinib once the metastatic disease was identified. From the patients’ records, three of the five patients had incidentally detected thyroid mass. The other two patients had known thyroid nodules that were monitored with serial ultrasounds and enlargement prompted the biopsy, which identified the metastatic disease.

FNAs are commonly used as a screening tool to separate between benign and malignant thyroid nodules. They allow the pathologist a glimpse into the cellular components of nodular proliferation, which, due to significant morphologic overlap between multiple entities, will be further interpreted in the patient's clinical context. The sample size is always a consideration and inadequate specimens may not provide the clinicians with a diagnosis; therefore, surgical resection might be necessary to further characterize the neoplasm.

The most common primary sites of metastatic tumors to the thyroid are the lung, kidney, head and neck, and breast, with only occasional reports of colorectal adenocarcinoma (3). It has previously been noted in other studies that the time from diagnosis of RCC to metastasis to the thyroid gland was significant at an average of 106–113 months (2, 4).

Up to 50% of patients will present without a previous malignant diagnosis, but if known this information should be included with the clinical history of the requisition submitted with the specimen. Unfortunately, there are mimickers of primary tumors making the diagnosis difficult at times. In one of the five cases reviewed, there was a ThinPrep slide that showed small groups of cells that resembled Hurtle cells arranged in small groups and microfollicules; one of the mimics of CC-RCC is a follicular or Hurthle cell neoplasm. The cellblock, in that case, showed the neoplastic cells there were PAX8 and CD10 positive, PTH and TTF-1 negative, supporting a diagnosis of metastatic CC-RCC.

There have been other reports of RCC metastatic to the thyroid from around the world but due to the low incidence, most studies include only a small number of subjects. The largest study reports thirty-six cases, this was completed by Heffess et al. and they reported comparable results to our study with a mean time to recurrence of 9.4 years but with a maximum time of 21.8 years (5). As noted in Table 2, other case reports discuss findings at various times from the initial diagnosis of primary tumor ranging from 6 months to 19 years. Similar to these case report studies our study is limited by a small sample size at a single institution. There is a need for continued reports as this is a rare disease, which is difficult to diagnose and can occur at a wide range of times after a patient initial diagnosis of renal cell carcinoma.

**Table 2:** Literature review with noted time to metastasis of renal cell carcinoma to the thyroid.

Study	Number of participants	Average age (years)	Average time to metastasis (years)
Heffess et al.	36	64.9	9.4
Benoit et al.	7	66	3.2
Muramoto et al.	1	54	5
Uzel	1	45	8
Kitamura et al.	1	63	0.5
Wada et al.	1	77	19
Ozdemir et al.	1		11
Zahradka et al.	1	80	11
de Lima et al.	1		17

## Conclusion

Patients with a history of renal cell carcinoma presenting with a thyroid nodule should be considered for the possible sites of metastatic disease no matter the time frame from initial diagnosis. Increased awareness and a high index of suspicion are needed to detect metastasis, as they can be found in atypical locations and mimic primary disease.

## Data Availability

The raw data supporting the conclusions of this article will be made available by the authors, without undue reservation.

## References

[1] Shabsigh A Sourial M Bellows FF McClung C Jayanthi R Kielb S Urology. In: Brunicardi F Andersen DK Billiar TR Dunn DL Kao LS Hunter JG, editors. Schwartz’s principles of surgery, 11e. McGraw-Hill (2019). Available from: https://accesssurgery.mhmedical.com/content.aspx?bookid=2576&sectionid=216211705

[2] Motzer RJ. Renal cell carcinoma. In: Jameson J Fauci AS Kasper DL Hauser SL Longo DL Loscalzo J, editors. Harrison’s principles of internal medicine, 20e. McGraw Hill. (2022). Available from: https://accessmedicine-mhmedical-com.gsmezproxy.utmck.edu/content.aspx?bookid=2129&sectionid=192016249 (Accessed August 11, 2021).

[3] Nakamura K Nozawa K Aoyagi Y Ishihara S Matsuda K Fukushima J A case report of thyroid gland metastasis associated with lung metastasis from colon cancer. Tumori. (2011) 97:229–32. 10.1177/030089161109700217 21617721

[4] Nakhjavani MK Gharib H Goellner JR van Heerden JA. Metastasis to the thyroid gland. A report of 43 cases. Cancer. (1997) 79:574–8. 10.1002/(SICI)1097-0142(19970201)79:3<574::AID-CNCR21>3.0.CO;2-# 9028370

[5] Heffess CS Wenig BM Thompson LD. Metastatic renal cell carcinoma to the thyroid gland: a clinicopathologic study of 36 cases. Cancer. (2002) 95(9):1869–78. 10.1002/cncr.10901 12404280

